# Effect of admission fascia iliaca compartment blocks on post-operative abbreviated mental test scores in elderly fractured neck of femur patients: a retrospective cohort study

**DOI:** 10.1186/s12871-016-0297-8

**Published:** 2017-01-05

**Authors:** Peter M. Odor, Irina Chis Ster, Iain Wilkinson, Frederic Sage

**Affiliations:** 1 Perioperative Medicine Fellow, University College London Hospital, London, UK; 2 Senior Lecturer in Biostatistics, Institute of Infection and Immunity, St. George’s University of London, London, UK; 3 Consultant, Orthogeriatrics, East Surrey Hospital, Redhill, Surrey UK; 4 Consultant, Department of Anaesthesia, East Surrey Hospital, Redhill, Surrey UK

**Keywords:** Fascia iliaca nerve block, Ageing: CNS changes, POCD: predictors, Opioids and ageing

## Abstract

**Background:**

Post-operative cognitive impairment is common in elderly patients following surgery for hip fracture, with undertreated pain being an important etiological factor. Non-opioid based analgesic techniques, such as nerve blocks, may help reduce the risk of cognitive complications. The aim of this study was to investigate whether receiving a fascia iliaca compartment block (FICB) as part of a pre-operative analgesic regime increased the odds of high post-operative abbreviated mental test scores (AMTS) when compared with conventional analgesia without a nerve block.

**Methods:**

A retrospective data analysis of a cohort of 959 patients, aged ≥ 65 years with a diagnosis of hip fracture and admitted to a single hospital over a two-year period was performed. A standardized analgesic regime was used on all patients, and 541/959 (56.4%) of included patients received a FICB. Provision of the FICB was primarily determined by availability of an anesthetist, rather than by patient status and condition. Post-operative cognitive ordinal outcomes were defined by AMTS severity as high (score of ≥9/10), moderate, (score of 7–8) and low (score of ≤6). A multivariable ordinal logistic regression analysis was performed on patient status and clinical care factors, including admission AMTS, age, gender, source of admission, time to surgery, type of anesthesia and ASA score.

**Results:**

Admission FICB was associated with higher adjusted odds for a high AMTS (score of ≥9) relative to lower AMTS (score of ≤8) than conventional analgesia only (OR = 1.80, 95% CI 1.27–2.54; *p* = 0.001). Increasing age, lower AMTS on admission to hospital, and being admitted from a residential or nursing home were associated with worse cognitive outcomes. Mode of anesthesia or surgery did not significantly influence post-operative AMTS.

**Conclusion:**

Post-operative AMTS is influenced by pre-operative analgesic regimes in elderly patients with hip fracture. Provision of a FICB to patients on arrival to hospital may improve early post-operative cognitive performance in this population.

## Background

Undertreated pain and inadequate analgesia are important risk factors for development of post-operative cognitive impairment and delirium in patients following hip fracture [1]. Not only is cognitive impairment common in patients suffering hip fractures [1], but the pathology has severe implications for the perioperative management and rehabilitation of such patients, leading to a longer length of hospital stay and greater mortality. Cognitive impairment encompasses a spectrum of conditions, including acute delirium and chronic dementia. Irrespective of etiology, cognitive impairment in patients following a hip fracture can adversely affect the patients’ ability to self-care, independently mobilize, request nursing assistance and engage with other post-operative recovery processes. Post-operative delirium in elderly patients is also associated with increased one year mortality and worse functional outcomes [2]. Hence, early intervention to reduce the risk of cognitive decline in patients with hip fracture represents a clear opportunity to improve post-operative outcomes and recovery.

Fascia iliaca compartment nerve blocks (FICB) provide opioid-sparing analgesia to patients with hip fracture [3], which helps mitigate against the severe pain [4] caused by the underlying pathology. Provision of effective analgesia may reduce the risk of developing post-operative cognitive impairment and delirium [5]. The aim of this study was to investigate whether patients receiving FICB were more likely to exhibit high post-operative abbreviated mental test scores (AMTS) than those patients who received conventional analgesia without a nerve block.

## Methods

Institutional approval by the R&D department and Caldicott Guardian were obtained for this retrospective data analysis of a cohort of patients diagnosed with hip fracture. The research was limited to secondary use of data previously collected during routine clinical care. Data collection was originally intended for national and local clinical audit purposes; accordingly, the requirement for further ethical approval was waived. All patient identifiers were anonymized before entering the data analysis process. All data, including abbreviated mental test scores, was collected in accordance with standard requirements provided by the UK Department of Health for hip fracture patients.

FICB have been routinely provided for analgesia in patients with a diagnosis of hip fracture at East Surrey Hospital, UK since 2010. FICB are single injection nerve blocks, involving the introduction of local anesthetic via a superficial needle in the groin into the fascia iliaca compartment, from where local anesthetic spreads to the femoral, lateral cutaneous and obturator nerves. Although the distribution of nerve blockade depends upon injection technique and the extent of local anesthetic spread, analgesia of the medial and lateral thigh can be consistently achieved [6]. All FICBs at East Surrey Hospital are performed by anesthetists following admission of patients with hip fracture into the local Emergency Department and in accordance with a standardized dosing regime. This regime involves an initial bolus dose of 30mls of 0.25% levobupivacaine, followed by introduction of a 18G catheter and subsequent infusion of 7 ml/h 0.125% levobupivacaine into the fascia iliaca compartment. During the date range used for data analysis FICB were performed at a median time of 6:15 [4:30–9:48 (0:16–73.6)] hours following patient arrival at the Emergency Department.

All patients presenting with a diagnosis of hip fracture between 1st July 2012 to 30th June 2014 were included in the data analysis. Individual patient records were excluded if the patient age was <65 years or if no surgery for hip fracture was conducted. The dataset used for retrospective analysis included patient characteristics, details of FICB performance and Abbreviated Mental Test Scores (AMTS) [7], all of which were collected as part of routine clinical care monitoring. The primary determinant of FICB allocation throughout the period was availability of an anesthetist to provide the service, rather than an informative choice based upon patient factors or contraindications to provision of the nerve block, of which there are few. Any related bias was minimized by conducting a multivariable analysis. There was a near even split between patients who received a FICB and those that did not, with 541/959 (56.4%) patients receiving FICB. All patients were prescribed a standardized regime of regular paracetamol, alongside pro re nata codeine and oramorph for analgesic purposes.

Patient data included was age, gender, American Society of Anesthesiologists (ASA) rating scale, source of admission, time to surgery and type of anesthesia. Abbreviated Mental Test Scores (AMTS) [7] were recorded in accordance with standard requirements provided by the UK Department of Health for hip fracture patients. AMTS is a 10 point score allocated according to the verbal responses to 10 questions (see Appendix 1), which has been validated for detecting cognitive impairment in geriatric populations [8]. Given non-linearity of the AMTS scale, values were categorized for analysis purposes to obtain ordinal outcomes accordingly: high for scores 9–10, moderate for 7–8 and low for ≤ 6. AMTS categories were pragmatically selected to conform with clinically relevant thresholds for detection of delirium [9, 10]. Ordinal logistic regression was employed to assess the univariate associations of post-operative AMTS with all the variables of interest, based on complete observations only. Further multivariable analyses were conducted to determine the most parsimonious model based on the available data – the model with the least number of predictors, yet explaining the most data variability. The adjusted associations between the post-operative AMTS outcome and the rest of the variables were quantified by the odds ratio of a high AMTS category versus all lower categories. Models’ appropriateness was checked using the Brant test for proportional odds (parallel regression) assumption. Hence the coefficients that describe the relationship between the lowest versus all higher categories of the response variable are the same as those that describe the relationship between the next lowest category and all higher categories. Patterns in any missing AMTS data were investigated in relationship to the outcome. An observed data analysis was further conducted under missing at random assumption (MAR) using multiple imputation techniques [11]. The estimates and their precision did not present with any substantial change, hence the final inference is the result of this MAR data analysis.

## Results

A total of 959 patients were included in the analysis. The proportion of patients who received FICB on admission to hospital was 541/959 (56.4%). From a total data set of 959 patients, 934 (97.4%) had a pre-operative AMTS recorded and 909 patients (94.8%) had a post-operative AMTS recorded. A total of 890 patients (92.8%) had both pre- and post-operative AMTS recorded. 113/959 (11.7%) patients were assessed for FICB by an anesthetist but the procedure was not performed due to patient factors (including disordered coagulation, patient refusal and lack of cooperation).

Patients were predominantly female (male:female ratio = 1:1.39) and elderly, with a mean (SD) age of 83.4 (7.9) years. Patient characteristics categorized according to post-operative AMTS are shown in Table 1. Overall the proportion of patients categorized as having a high AMTS (AMTS ≥9) decreased between admission and post-operative period, with 504/934 (54.0%) patients scoring high on admission, compared with 434/909 (45.3%) patients post-operatively. Pre-operative AMTS category was significantly associated with post-operative AMTS category, as was age and source of admission (all *p* < 0.01).

**Table 1:** Characteristics of participants according to post-operative abbreviated mental test score

	Low post op AMTS (*n* = 336)^a^	Moderate post op AMTS (*n* = 139)^a^	High post op AMTS (*n* = 434)^a^
Age; years	89.8 (7)	84.3 (6.8)	81.3 (8.2)
Admission AMTS			
High	22 (6.5%)	64 (46.0%)	392 (90.3%)
Moderate	26 (7.7%)	32 (23.0%)	24 (5.5%)
Low	286 (85.1%)	37 (26.6%)	11 (2.5%)
Gender			
M	81 (24.1%)	43 (30.9%)	127 (29.3%)
F	255 (75.9%)	96 (69.1%)	307 (70.7%)
Source of admission			
Own home	135 (40.2%)	119 (83.8%)	405 (93.3%)
Residential home	99 (29.5%)	9 (6.3%)	10 (2.3%)
Nursing home or hospital	102 (30.3%)	11 (9.9%)	19 (4.3%)
Comorbities			
ASA 1–2	59 (17.6%)	49 (35.3%)	218 (50.5%)^b^
ASA 3	235 (69.9%)	78 (56.1%)	191 (44.2%)
ASA 4–5	42 (12.5%)	12 (8.6%)	23 (5.3%)
Type of anesthesia			
GA	292 (87.4%)	11 (79.9%)	346 (79.7%)
Spinal	33 (9.9%)	26 (18.7%)	75 (17.3%)
GA + spinal	9 (2.7%)	2 (1.4%)	13 (3.0%)
Time to surgery; hours	21.6 (16.6–29.1 [5–670])	21.7 (15.9–27.2 [5–140])	21.0 (16.3–28.2 [1–230])
Received admission fascia iliaca compartment block			
Yes	161 (47.9%)	79 (56.8%)	284 (65.4%)
No	175 (52.1%)	60 (43.2%)	150 (34.6%)

Values are expressed as number (proportion), or median (IQR [range])

^a^A total of 909 patients had post-operative AMTS recorded, from the total of all 959 patients included in the analysis

^b^2/434 patients with High post op AMTS did not have ASA recorded pre-operatively

Variables included in the multivariable logistic regression model included: receipt of an admission FICB, admission AMTS, age, source of admission, ASA, mode of anesthesia and time to surgery (log). After entry into the multivariable logistic regression model the adjusted proportional odds ratio (OR) of scoring a high post-operative AMTS (score of ≥9) versus ≤8 was 1.80 (95% CI 1.27–2.54) for patients receiving FICB compared with conventional analgesia only (*p* = 0.001). The adjusted ORs for high, moderate and low post-operative AMTS outcomes in relation to the important explanatory variables in the final regression model are described in Tables 2 and 3. Admission AMTS was highly predictive of post-operative AMTS. The odds for retaining a high pre-operative AMTS into the post-operative period were significantly higher than for moderate pre-operative AMTS patients increasing to high AMTS post-operatively (OR = 9.79, 95% CI 6.0–15.9; *p* < 0.001). Increasing age reduced the likelihood of scoring a high post-operative AMTS, such that an increment in age by an additional 5 years reduces the OR for post-operative AMTS being high versus ≤8 to 0.85 (95% CI 0.76–0.96). Gender, mode of anesthesia and comorbidity status (measured using the ASA scale) did not have any statistically significant effect on post-operative AMTS outcomes. Patients admitted to hospital from a care home had significantly lower odds of having a high post-operative AMTS than those admitted from their own homes, even when adjusted for other potential confounders (OR = 0.35, 95% CI 0.17–0.65; *p* = 0.001). As the time delay for surgery increased on a logarithmic scale, the OR for a high post-operative AMTS reduced to below 1.0, although this association only marginally met predefined statistically significant limits (*p* = 0.05).

**Table 2:** Univariate analysis of predictors of post-operative high AMTS, compared with moderate or low AMTS

	Proportional odd ratios	*p* value	Odd ratio lower bound (95%)	Odds ratio upper bound (95%)
Received admission fascia iliaca compartment block				
Yes vs. No	1.89	<0.001	1.34	2.66
Admission AMTS				
High vs. Low	130.7	<0.001	83.1	205.6
High vs. Moderate	10.6	<0.001	6.6	17.0
Moderate vs. Low	12.4	<0.001	7.4	20.7
Age at event				
1 year increment	0.97	0.011	0.95	0.99
5 year increment	0.86	0.011	0.77	0.97
10 year increment	0.74	0.011	0.59	0.93
Age (log)	0.09	0.010	0.01	0.55
Gender				
Female vs. male	1.14	0.49	0.79	1.66
Source of admission				
Residential vs. own home	0.30	<0.001	0.14	0.54
Nursing home or hospital vs. own home	0.50	0.016	0.29	0.88
Nursing home or hospital vs. residential	1.80	0.14	0.83	3.91
Comorbities				
ASA 3 vs. ASA 1–2	0.72	0.09	0.50	1.05
ASA 4–5 vs. ASA 1–2	0.44	0.017	0.23	0.86
ASA 4–5 vs. ASA 3	0.61	0.13	0.32	1.16
Mode of anesthesia				
GA + spinal vs. GA	0.57	0.28	0.21	1.59
Spinal vs. GA	1.21	0.43	0.75	1.96
Spinal vs. GA + spinal	2.12	0.18	0.71	6.35
Time to surgery	0.99	0.44	0.99	1.00
Time to surgery (log)	0.74	0.06	0.54	1.02
Length of stay	0.98	<0.001	0.97	0.99
Length of stay (log)	0.58	<0.001	0.45	0.75
Nottingham Hip Fracture Score (NHFS)	0.82	0.009	0.71	0.95
Type of surgery				
Dynamic hip screw vs. arthroplasty	1.03	0.94	0.45	2.35
Intramedullary nailing vs. arthroplasty	1.23	0.25	0.86	1.73
Dynamic hip screw vs. intramedullary nailing	1.19	0.68	0.52	2.72

An odds ratio (OR) > 1 represents a higher probability of the primary outcome measure (post-operative AMTS ≥9 versus ≤8) according to the reference category

**Table 3:** Multivariable ordinal logistic regression analysis of predictors of post-operative high AMTS, compared with moderate or low AMTS

	Proportional odd ratios	*p* value	Odd ratio lower bound (95%)	Odds ratio upper bound (95%)
Received admission fascia iliaca compartment block				
Yes vs. No	1.80	0.001	1.27	2.54
Admission AMTS				
High vs. Low	82.1	<0.001	49.7	135.7
High vs. Moderate	9.8	<0.001	6.0	15.9
Moderate vs. Low	8.4	<0.001	4.9	14.5
Age at event				
1 year increment	0.97	0.008	0.95	0.99
5 year increment	0.85	0.008	0.76	0.97
10 year increment	0.72	0.008	0.57	0.92
Source of admission				
Residential vs. own home	0.35	0.002	0.18	0.67
Nursing home or hospital vs. own home	0.56	0.04	0.32	0.96
Nursing home or hospital vs. residential	1.60	0.23	0.74	3.43
Comorbities				
ASA 3 vs. ASA 1–2	0.89	0.57	0.61	1.32
ASA 4–5 vs. ASA 1–2	0.53	0.06	0.27	1.03
ASA 4–5 vs. ASA 3	0.59	0.11	0.31	1.12
Mode of anesthesia				
GA + spinal vs. GA	0.52	0.24	0.17	1.55
Spinal vs. GA	1.39	0.20	0.84	2.29
Spinal vs. GA + spinal	2.70	0.09	0.83	8.8
Time to surgery (log)	0.72	0.05	0.52	1.00

An odds ratio (OR) > 1 represents a higher probability of the primary outcome measure (post-operative AMTS ≥9 versus ≤8) according to the reference category

## Discussion

Decline in post-operative cognitive function, delirium and persistent cognitive impairment are common problems in the hip fracture patient population, with a multifactorial etiology. Patients presenting with hip fracture have a high incidence of cognitive impairment (approximately 30%) and a further 15% typically become cognitively impaired post operatively [12]. Delirium is often under recognized in acute hospitals [13] so the true level may be even higher. This reduction in post-operative cognition is associated with impaired rehabilitation in hip fracture patients and with increased length of hospital stay [14]. Undertreated pain is an important etiological factor in post-operative cognitive impairment or delirium and furthermore pain following hip fracture has been specifically implicated in a range of perioperative complications that may extend hospital length of stay and time to operative intervention [15, 16]. Patients who sustain hip fractures often have a high degree of predisposing factors for post-operative cognitive dysfunction (such as pre-operative cognitive dysfunction, frailty and a high number of comorbidities [17], as in our patient group). Unfortunately, opioid analgesics are also commonly associated as a delirium precipitant.

FICB are already known to be appropriate for and advantageous to hip fracture patients; providing significant benefit in pain control, beginning within 15 min and lasting over eight hours following a single bolus injection [18]. Use of continuous infusion of local anesthetic into the fascia iliaca compartment, as was administered by default to patients involved in this study, has also been suggested to show patient outcome benefit following hip fracture [19]. Alongside these advantages FICB are simple to learn and their provision to hip fracture patients is consistent with guidance from NICE [20] and the AAGBI [21].

Our retrospective analysis evaluated early post-operative AMTS in patients admitted to a single hospital with hip fracture. After adjustment for confounding factors we found higher odds for high post-operative AMTS versus AMTS of ≤8 in patients receiving FICB compared with conventional analgesia (OR = 1.80, 95% CI 1.27–2.54; *p* = 0.001). Patients had a worse early cognitive outcome if they did not receive FICB, even when adjusted for admission AMTS, age, source of admission, ASA and time to surgery (log). These results suggest that the administration of pre-operative FICB may be independently associated with high post-operative AMTS in hip fracture patients. Serial testing of AMTS has been shown to be helpful in identifying acute cognitive dysfunction in the elderly [9], with a decline of two or more points having high sensitivity and specificity for diagnosing post-operative delirium [10].

A single randomized controlled trial has provided some evidence of FICB benefit in delirium prophylaxis for 207 patients with hip fracture [5]. Patients in this trial were randomized upon admission to hospital to receive FICB or placebo sham blocks. The FICB group had a decreased incidence of delirium (10% vs. 33%), alongside a reduced severity and a shorter duration of delirium. However, the study had significant risk of bias, showed insufficient allocation concealment and blinding, and was without an intention-to-treat analysis. The use of pethidine as a default analgesic in the control group is also controversial and may have favored an increased comparative incidence of delirium in control subjects. Similar results have been found in a quality improvement project looking at 41 patients who underwent FICB and 41 who did not in a single unit in the UK; here delirium and post-operative opioid usage were reduced in the FICB group [22].

Effective multimodal pain relief has been shown to reduce the incidence of post-operative delirium in prospective interventional studies in hip fracture patients [23, 24]. Our study adds to an increasing body of research literature that supports the use of FICB as a specific intervention, as part of a multimodal analgesic regime, to protect vulnerable hip fracture patients from the risk of developing post-operative cognitive impairment and delirium. Given the deleterious consequences of cognitive impairment and the low risk of FICBs, our results provide an additional incentive to establish effective methods of delivering this specific analgesic technique to hip fracture patients during the pre-operative period.

This study also demonstrated several additional observations regarding cognitive performance in hip fracture patients. As expected, pre-operative AMTS score was significantly associated with post-operative AMTS. Although most patients with a high admission AMTS retained high scores in the post-operative period, the odds of retaining a high AMTS were higher if a patient received a pre-operative FICB. The majority of patients with high post-operative AMTS were also admitted into hospital from their own homes, rather than residential care or a nursing home. Accordingly, a significant adjusted proportional odds ratio was found for comparisons between categories of admission AMTS and comparisons of source of admission to hospital for the outcome measure of a high post-operative AMTS. Patients with a high pre-operative AMTS had significantly higher odds of scoring a high post-operative AMTS than patients within the low or moderate category of pre-operative AMTS. Likewise patients admitted to hospital from a residential home had significantly lower odds of scoring a high post-operative AMTS than patients admitted from their own home. Gender, modality of anesthesia and measure of comorbidity status (using the ASA scale) were not found to have a statistically significant effect on post-operative AMTS outcomes. Although sufficiently powered high quality randomized studies of anesthesia modality in hip fracture are lacking, the majority of observational studies to date have failed to show major, significant outcome differences for spinal versus general anesthesia [25–29]. Our results of early cognitive function in hip fracture surgery patients are consistent with this trend.

The relationship between FICB and post-operative AMTS observed in this study does not prove causation. However, the results are concordant with other studies demonstrating that effective control of pain is important in preventing cognitive dysfunction in vulnerable geriatric patients with limited physiological reserve. The mechanism for this is not clear but we suggest the reduction in opioid usage and the potential reduction in localized pain may be key. The ordinal logistic regression model used in the study accounted for the most important, reliably recorded and readily available data parameters for hip fracture patients at the host institution. However, not all desired data was able to be included in the regression analysis. There are multiple chronic and acute factors that can predispose to cognitive impairment, including heart failure, anemia, malnutrition, stroke, polypharmacy and sepsis. Biochemical results, such as serum sodium concentrations, and markers of sepsis were not included in the regression model. Likewise, although widely used and relevant, AMTS alone is not the ideal diagnostic tool for delirium or for post-operative cognitive dysfunction (POCD). Further detailed clinical tests of delirium might have provided further support to the clinical validity of trends shown for AMTS in patient data included in our analysis.

## Conclusions

In conclusion, this retrospective cohort study provides supportive evidence for a beneficial effect of FICB in protecting against cognitive impairment in elderly patients with hip fracture. FICB may be an effective technique for reducing risk of delirium and the consequential effects this prevalent post-operative complication has within the increasingly important population of patients admitted to hospital with hip fracture.

## Appendix

### The Abbreviated Mental Test Score (AMTS) [7]

Questions (1 point for a correct response to each question):

1. What is your present age?
2. What is the time just now?
  Recall: Ask the patient to remember an address (e.g., 42 West Register Street)
3. What year is it?
4. What is our current location (e.g., name of hospital or town)?
5. Can you recognize these two people (e.g., relatives, carers or if none around, the likely profession of easily identified people such as doctor/nurse)?
6. What is your birthday?
7. What year did the Second World War begin?
8. What is the name of the current monarch or Prime Minister?
9. Please count sequentially backwards from 20 to 1.
10. Could you repeat the address which I gave you earlier?

## Abbreviations

AAGBI: Association of Anaesthetists of Great Britain and Ireland
AMTS: Abbreviated mental test score
ASA: American Society of Anesthesiologists
FICB: Fascia iliaca compartment block
MAR: Missing at random assumption
NICE: National Institute of Clinical Excellence
OR: Odds Ratio
POCD: Post-operative cognitive dysfunction
